# Supplementary material for: Reference quality assembly of the 3.5-Gb genome of Capsicum annuum from a single linked-read library
Source: Hortic Res. 2018 Jan 12;5:4. doi: 10.1038/s41438-017-0011-0 (PMC5798813; doi:10.1038/s41438-017-0011-0)
Supplement: Supplementary file 1 — Supplementary Figure [file 41438_2017_11_MOESM1_ESM.docx]

Supplemental Figures:


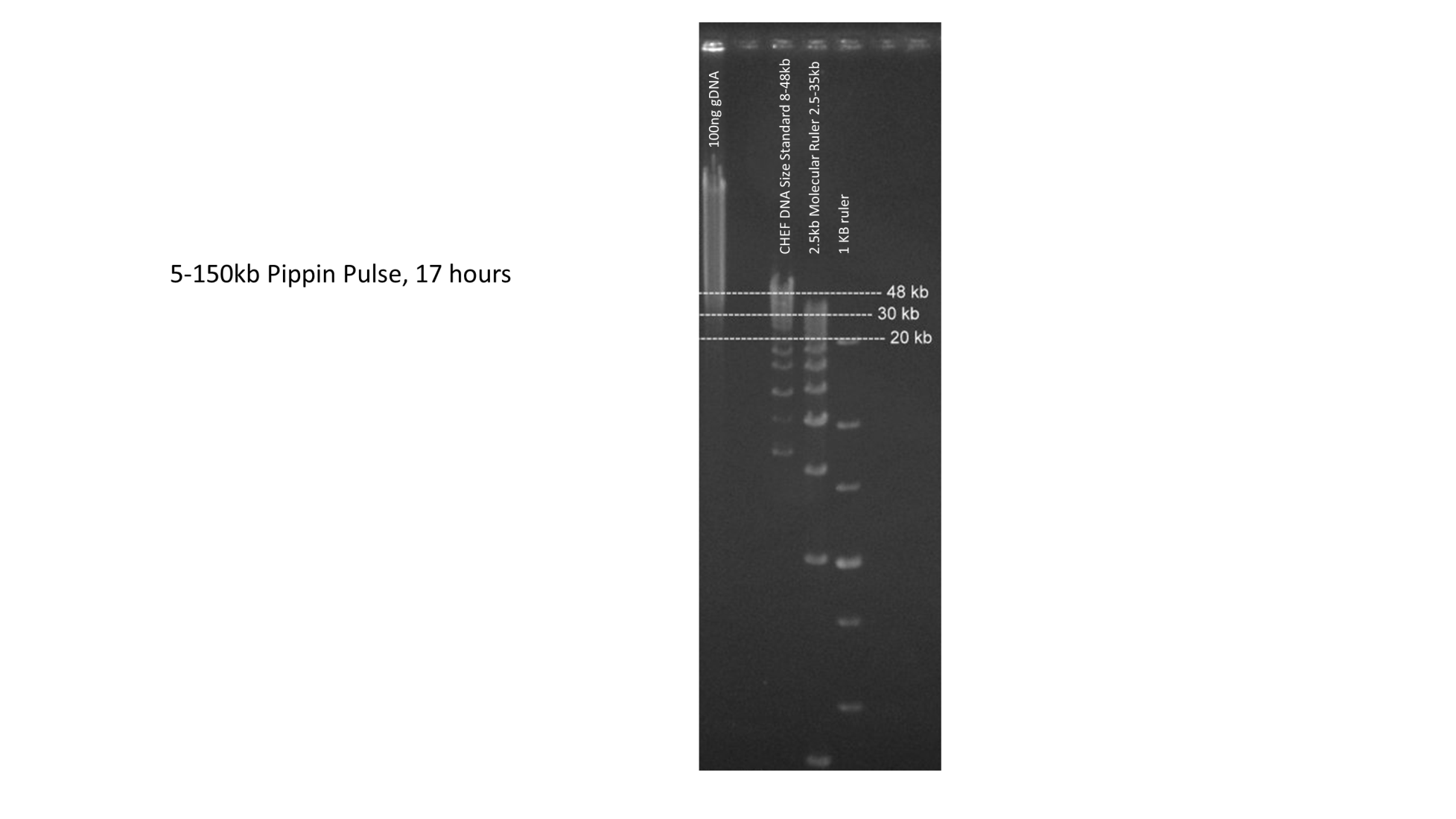


**Supplemental Figure 1 – Analysis of High-Molecular Weight DNA Extraction.** A pippen pulse field gel of 100 ng of genomic DNA from high-molecular weight DNA extraction of F1 pepper sample and three standard weight ladder samples run for 17 hours to show 5-150Kb molecule ranges.


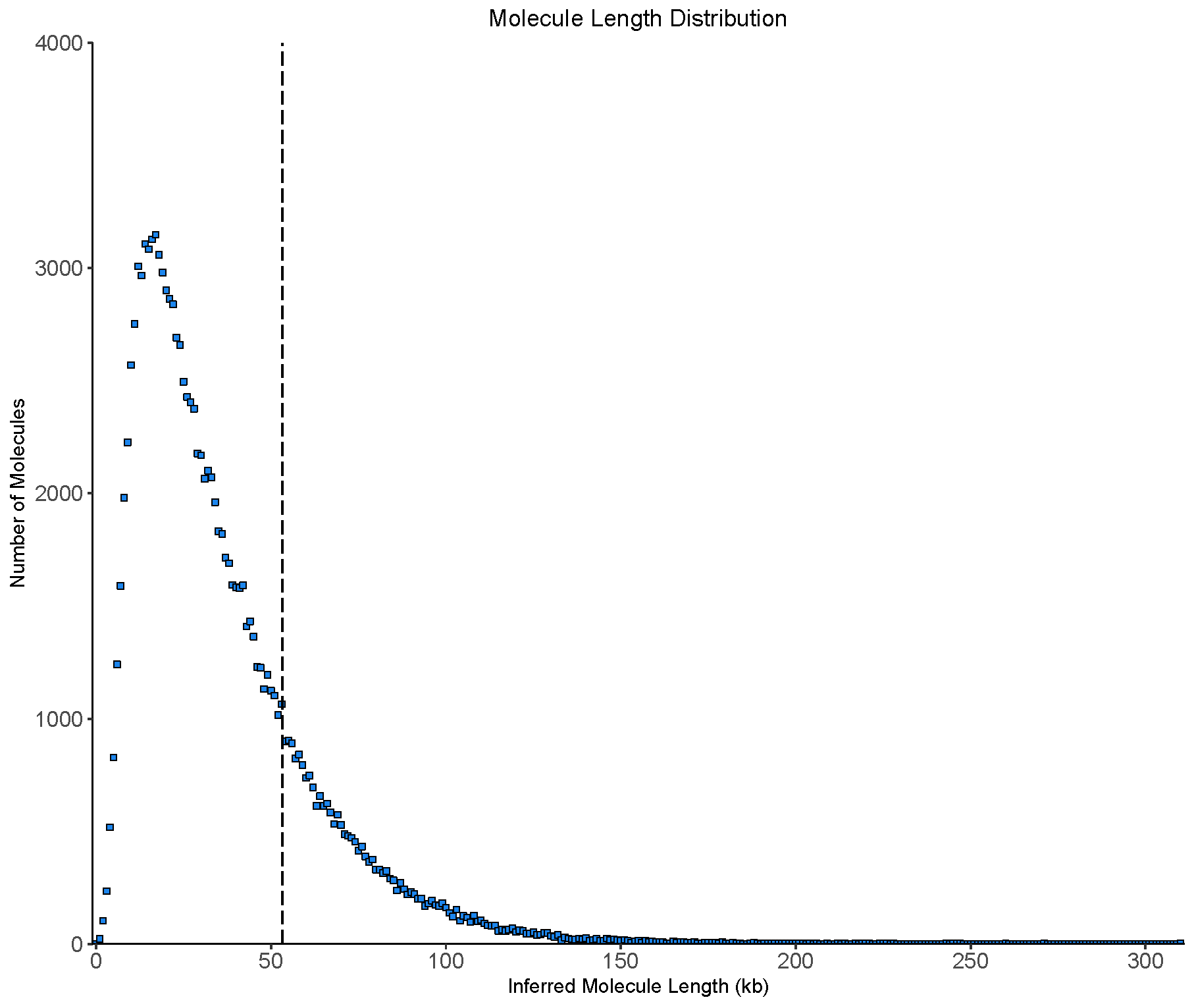


**Supplemental Figure 2 – Calculated Chromium Molecule Length Distribution.** Molecule length was calculated using the LongRanger tool through 10X Genomics based on the resulting raw assembly scaffolds. Weighted average molecule length is plotted at 53Kb with a dotted line.


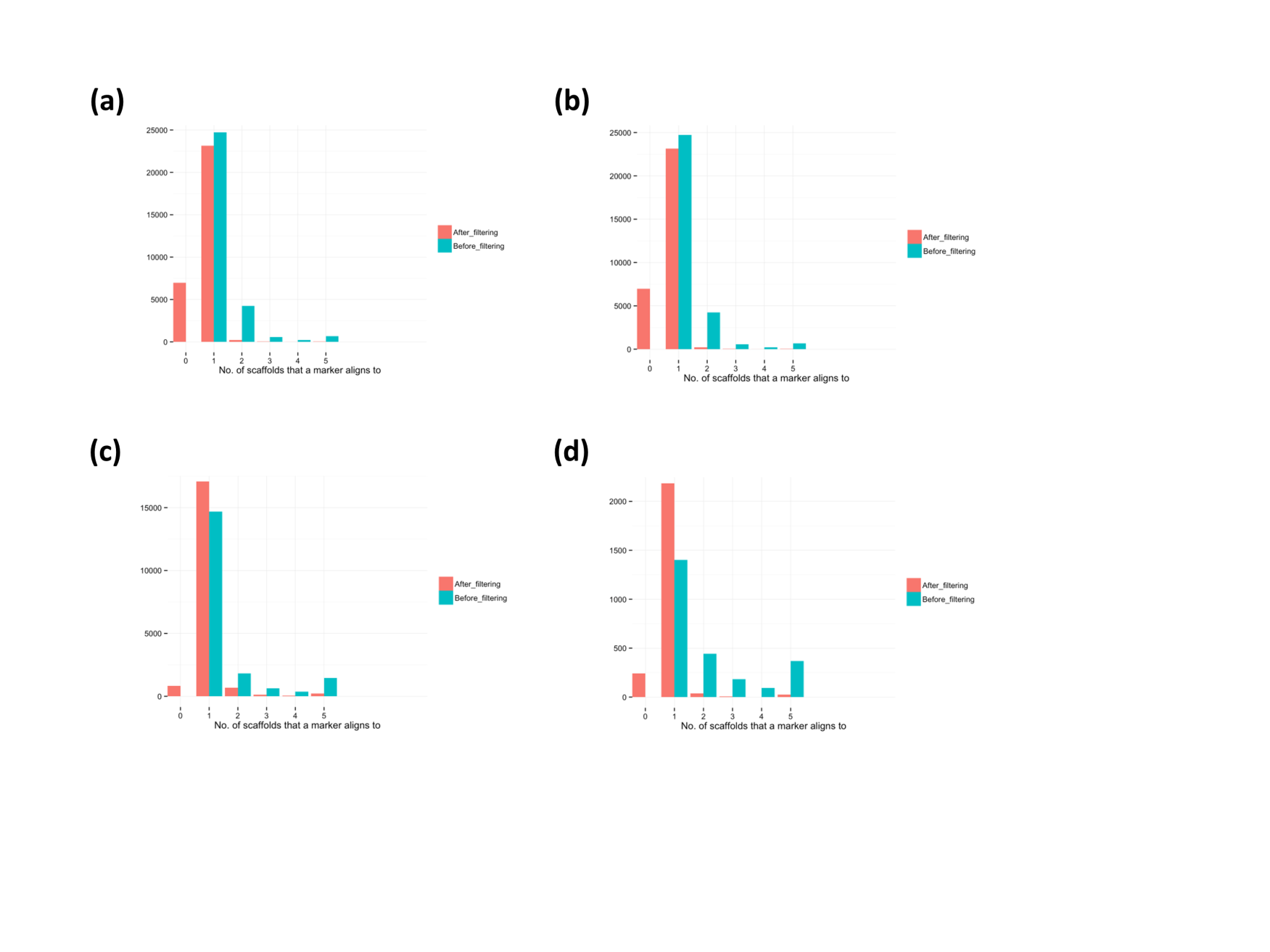


**Supplemental Figure 3 – Alignment Status of Markers to Raw Assembly Scaffolds.** Summary of the number of scaffolds that a marker aligned to on the 10X raw assembly scaffolds after filtering for (a) *C. frutescens* x *C. annuum* interspecific genetic map by Hill et al., (b) *C. annuum* x *C. annuum* intraspecific genetic map by Hill et al., (c) *C. frutescens* x *C. annuum* interspecific genetic map by Hulse-Kemp et al., and (d) *C. annuum* x *C. annuum* intraspecific genetic may by Han et al.


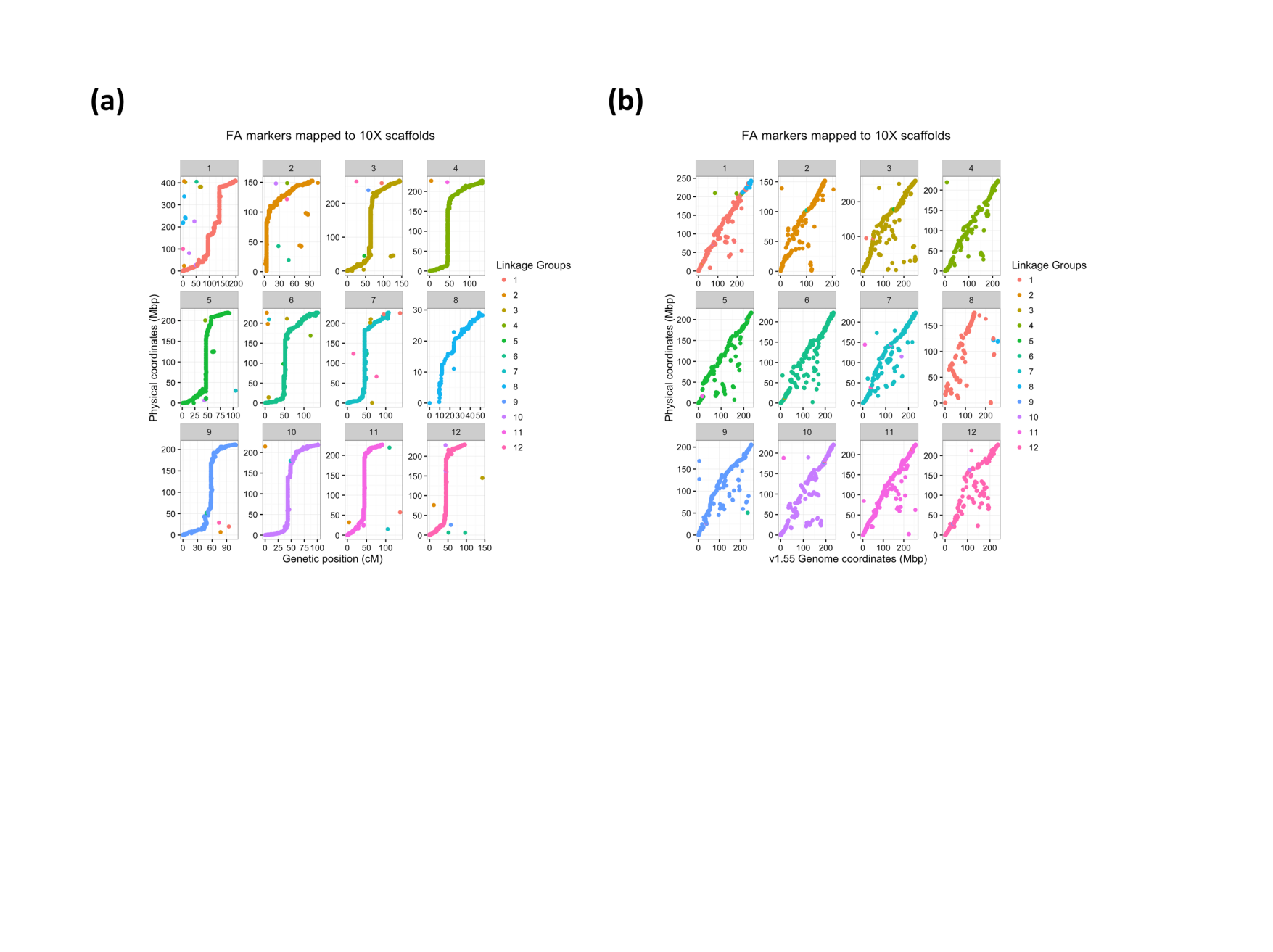


**Supplemental Figure 4 - Assessment of raw 10X assembly contigs compared to Hill et al. (2015)** *Capsicum frutescens* x *Capsicum annuum* genetic map, shown for all chromosomes. Assembly scaffolds are ordered based on their primary linkage group, sorted in order of increasing genetic distance. Linkage group colored labels correspond to marker linkage group in Hill et al. **A.**) Genetic positions of markers (centiMorgans) are shown versus the physical position on the 10X assembly contigs (megabase pair). **B.**) Physical position on the 10X assembly contigs (Mbp) versus physical position on the *C. annuum* CM334 version 1.55 assembly.


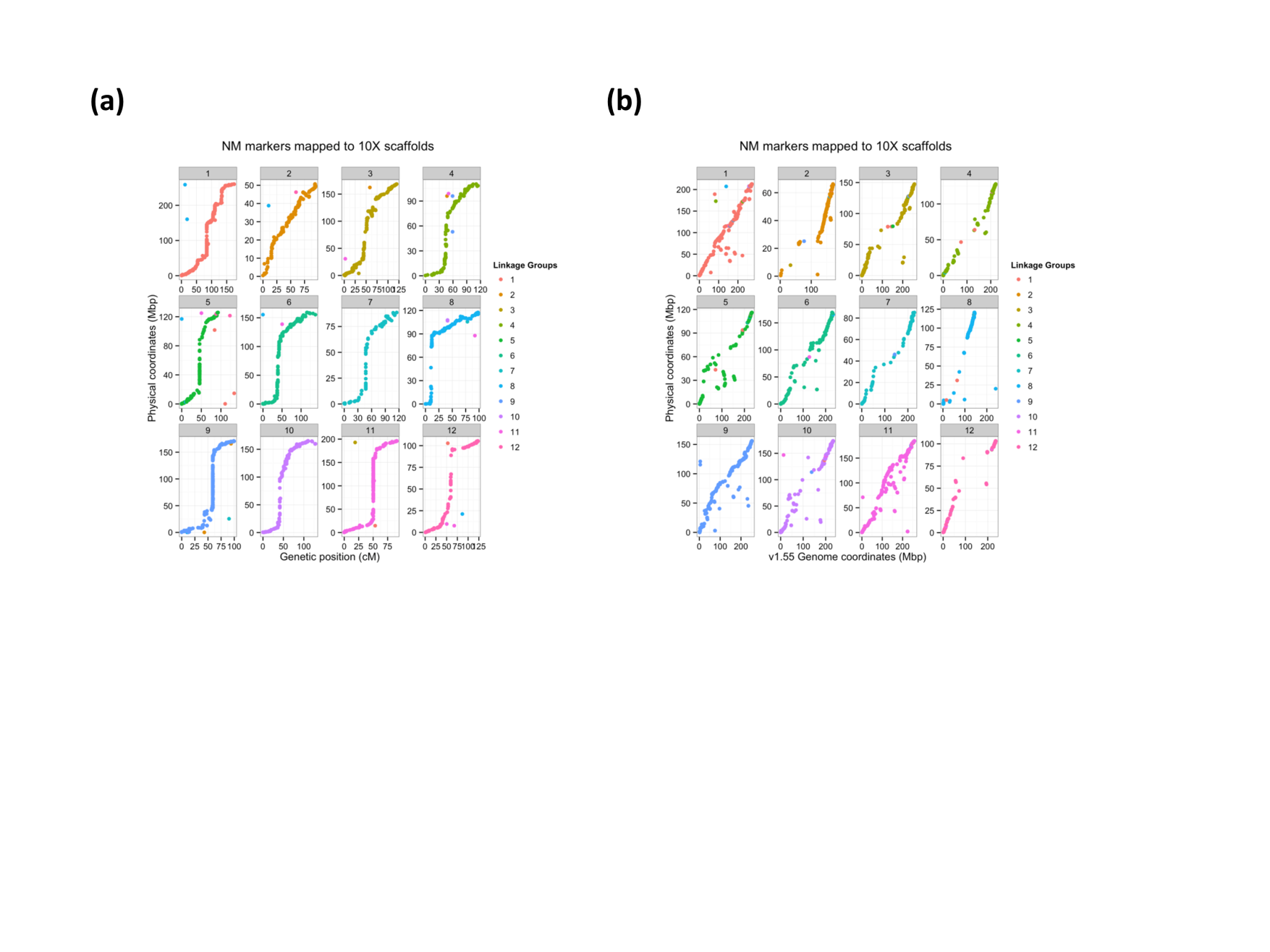


**Supplemental Figure 5 - Assessment of raw 10X assembly contigs** **compared to Hill et al. (2015)** *Capsicum annuum* x *Capsicum annuum* genetic map, shown for all chromosomes. Assembly scaffolds are ordered based on their primary linkage group, sorted in order of increasing genetic distance. Linkage group colored labels correspond to marker linkage group in Hill et al. **A.**) Genetic positions of markers (centiMorgans) are shown versus the physical position on the 10X assembly contigs (megabase pair). **B.**) Physical position on the 10X assembly contigs (Mbp) versus physical position on the *C. annuum* CM334 version 1.55 assembly.


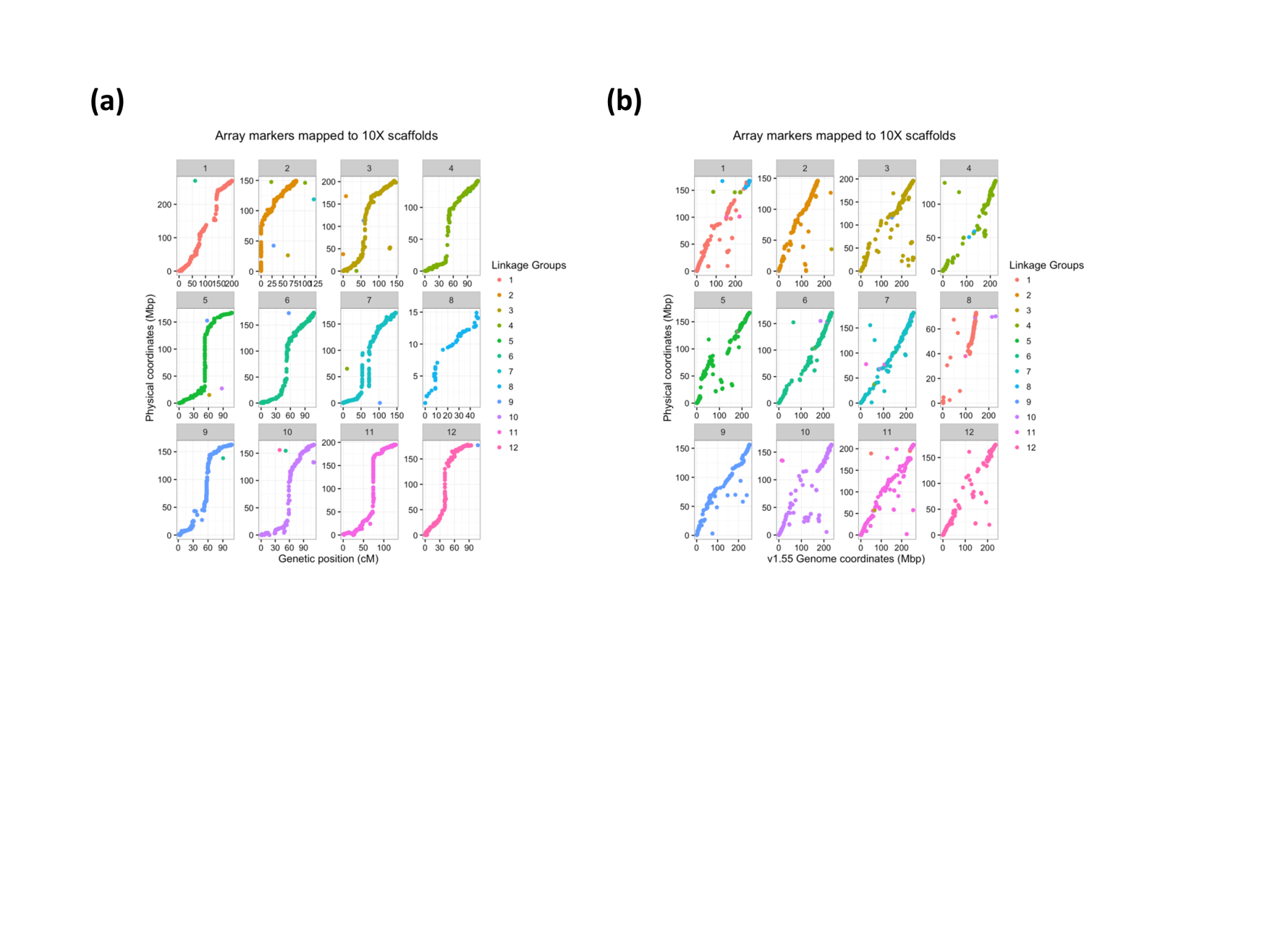


**Supplemental Figure 6 - Assessment of raw 10X assembly contigs** **compared to Hulse-Kemp et al. (2016)** *Capsicum frutescens* x *Capsicum annuum* genetic map, shown for all chromosomes. Assembly scaffolds are ordered based on their primary linkage group, sorted in order of increasing genetic distance. Linkage group colored labels correspond to marker linkage group in Hill et al. **A.**) Genetic positions of markers (centiMorgans) are shown versus the physical position on the 10X assembly contigs (megabase pair). **B.**) Physical position on the 10X assembly contigs (Mbp) versus physical position on the *C. annuum* CM334 version 1.55 assembly.


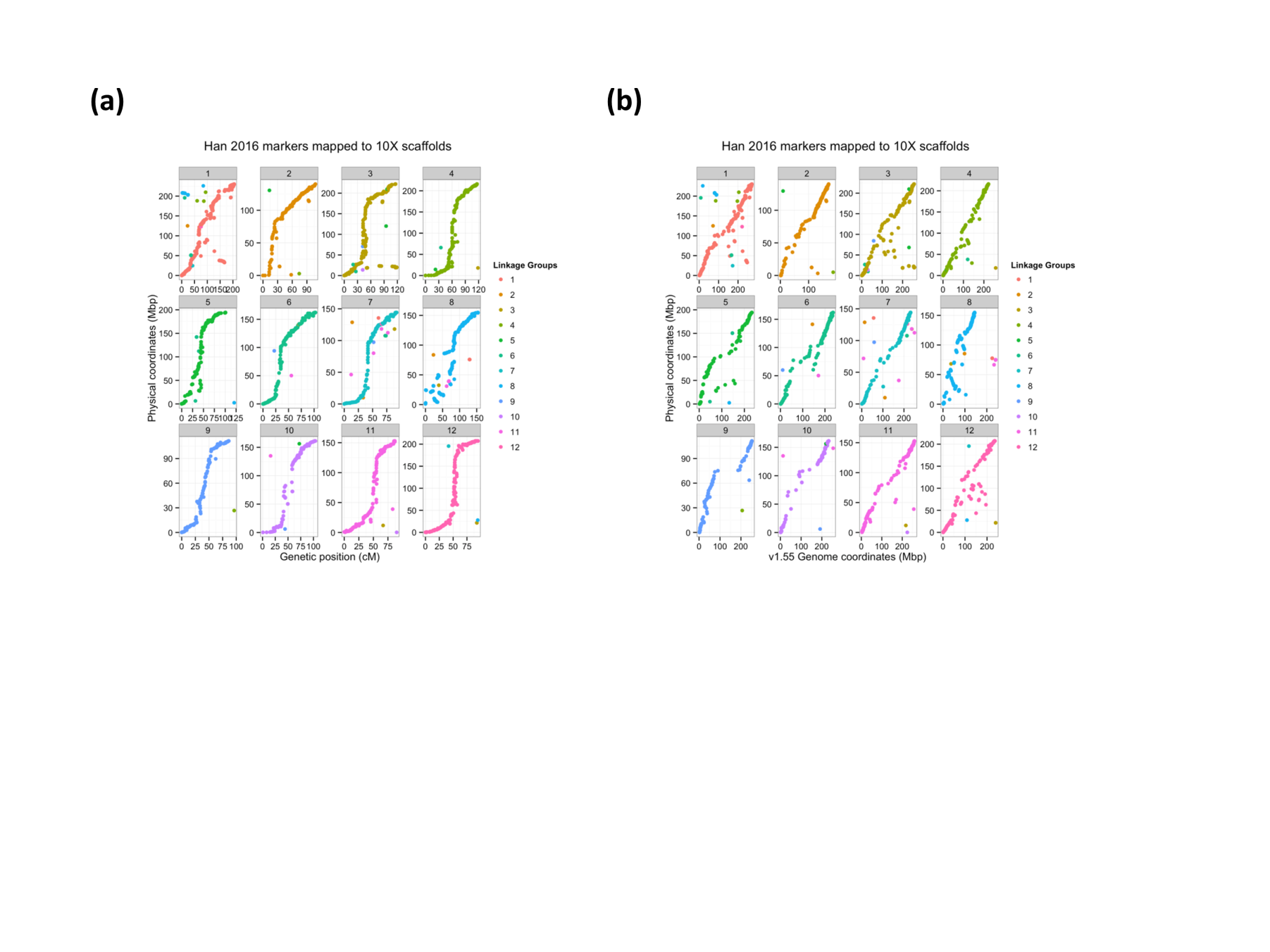


**Supplemental Figure 7 - Assessment of raw 10X assembly contigs** **compared to Han et al. (2016)** *Capsicum annuum* x *Capsicum annuum* genetic map, shown for all chromosomes. Assembly scaffolds are ordered based on their primary linkage group, sorted in order of increasing genetic distance. Linkage group colored labels correspond to marker linkage group in Hill et al. **A.**) Genetic positions of markers (centiMorgans) are shown versus the physical position on the 10X assembly contigs (megabase pair). **B.**) Physical position on the 10X assembly contigs (Mbp) versus physical position on the *C. annuum* CM334 version 1.55 assembly.


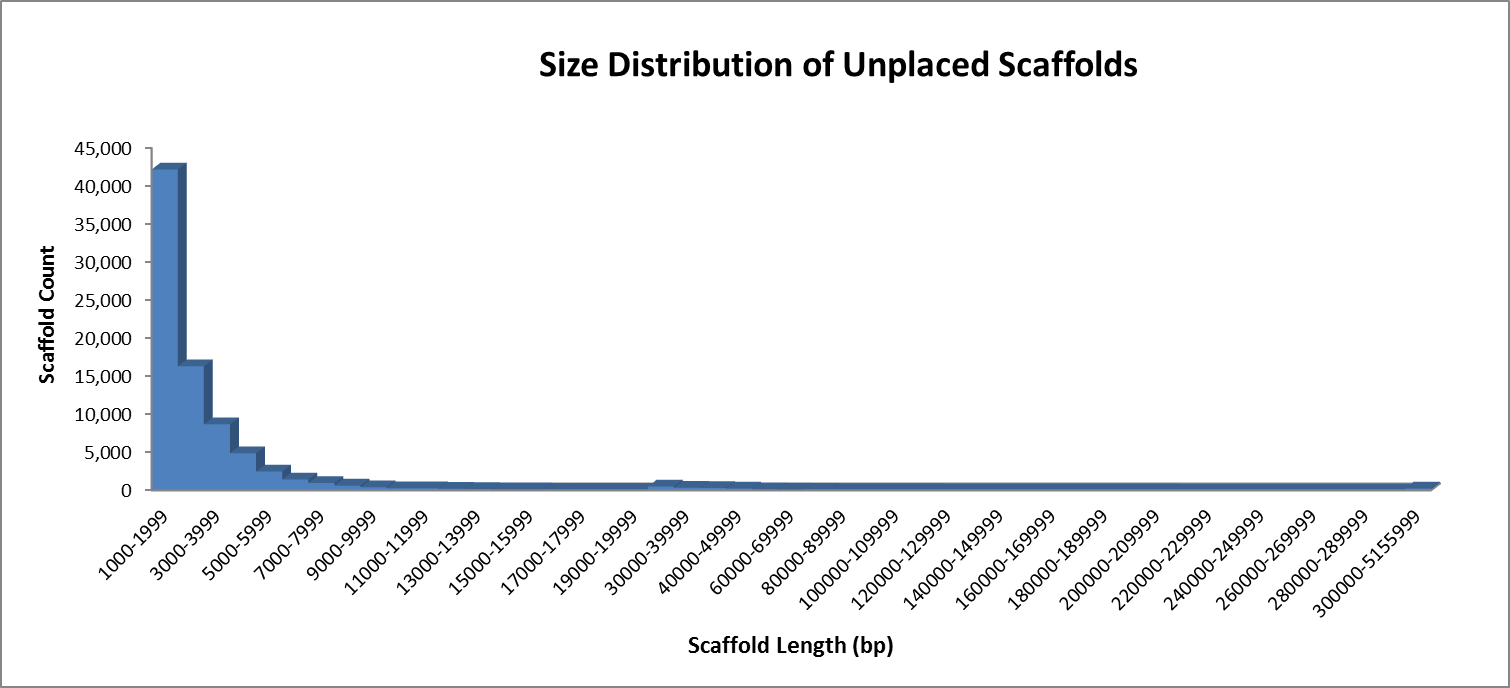


**Supplemental Figure 8 - Distribution of unplaced scaffold lengths.** The distribution of the lengths of unplaced scaffolds in the final UCD10X v1.0 *Capsicum annuum* assembly.


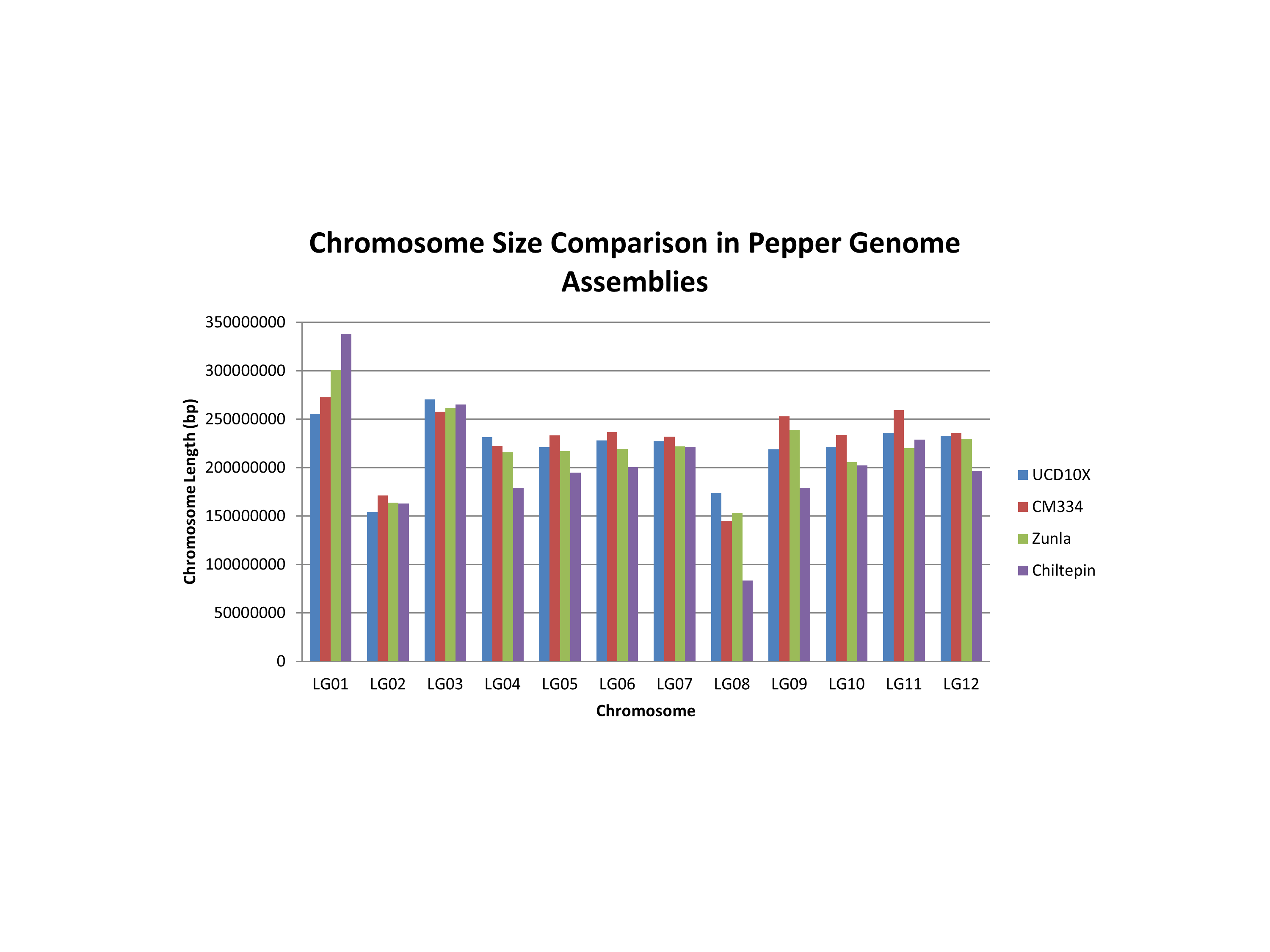


**Supplemental Figure 9 - Chromosome Size Comparison Between Pepper Genome Assemblies.** Size of chromosome pseudomolecule sequences from each of the four published pepper genome assemblies.


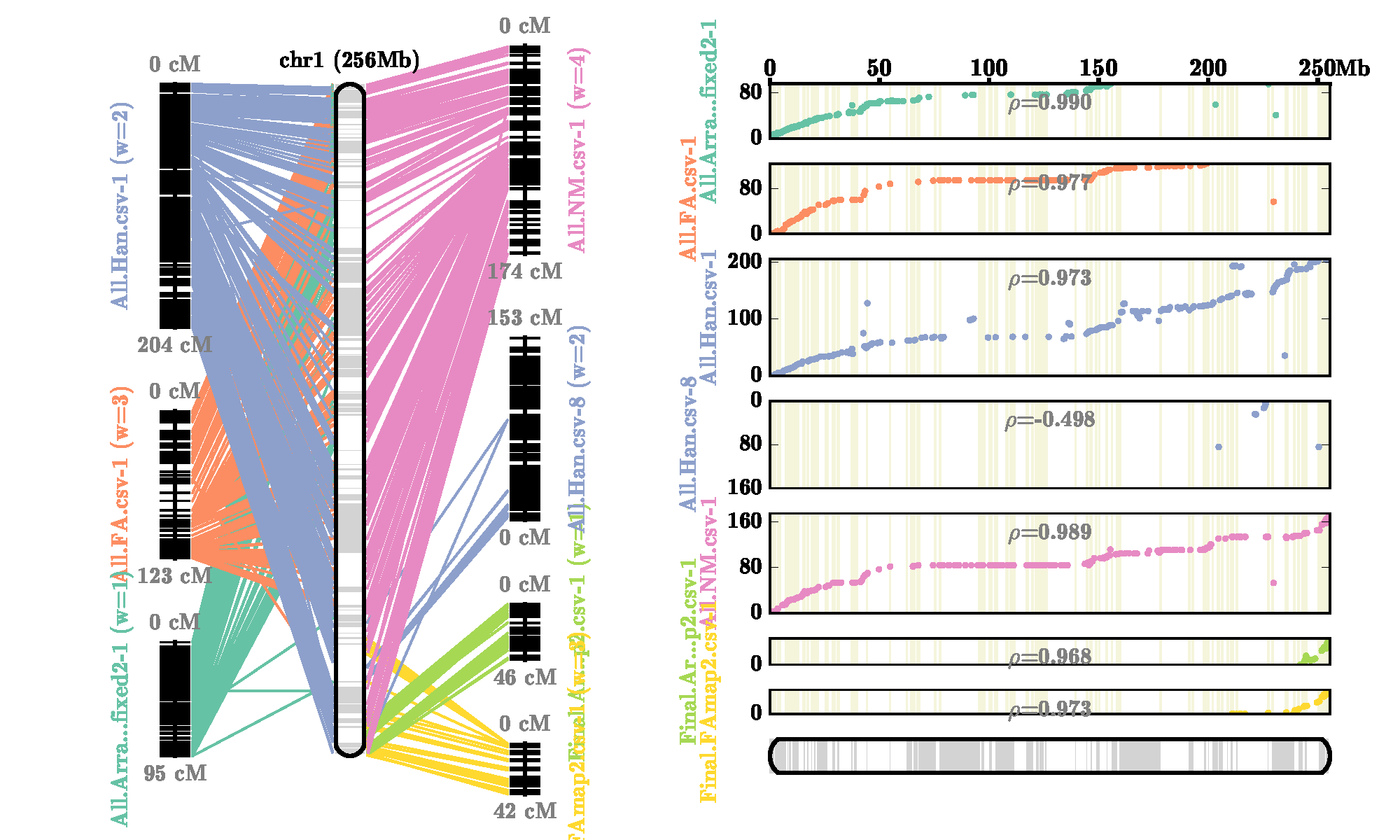


**Supplemental Figure 10 - AllMaps Chromosome 1 Consensus Maps for Pseudomolecule Generation.** Filtered alignment positions from Hill et al. (2015), Hulse-Kemp et al. (2016) and Han et al. (2017) were utilized to generate pseudomolecules using the AllMaps software.


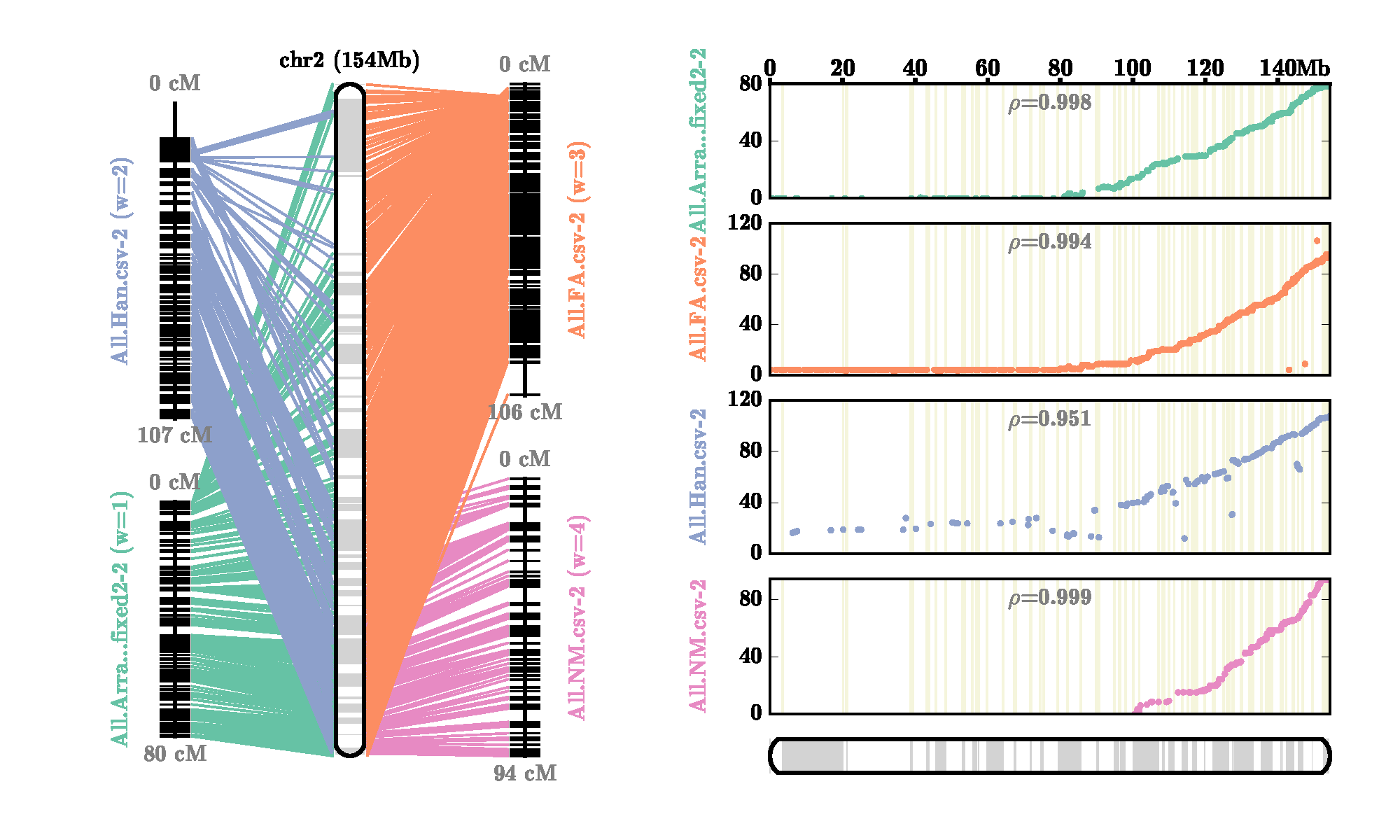


**Supplemental Figure 11 - AllMaps Chromosome 2 Consensus Maps for Pseudomolecule Generation.** Filtered alignment positions from Hill et al. (2015), Hulse-Kemp et al. (2016) and Han et al. (2017) were utilized to generate pseudomolecules using the AllMaps software.


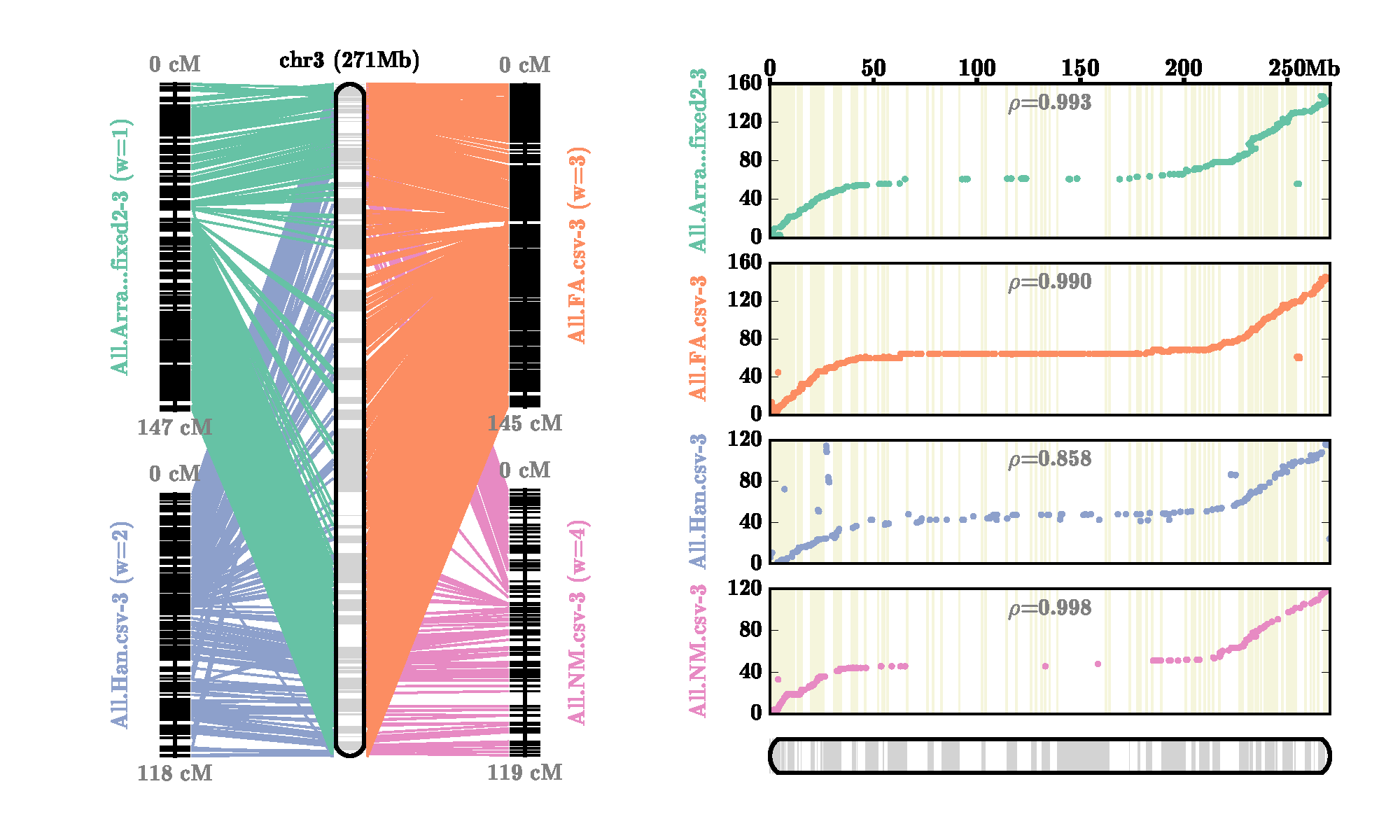


**Supplemental Figure 12 - AllMaps Chromosome 3 Consensus Maps for Pseudomolecule Generation.** Filtered alignment positions from Hill et al. (2015), Hulse-Kemp et al. (2016) and Han et al. (2017) were utilized to generate pseudomolecules using the AllMaps software.


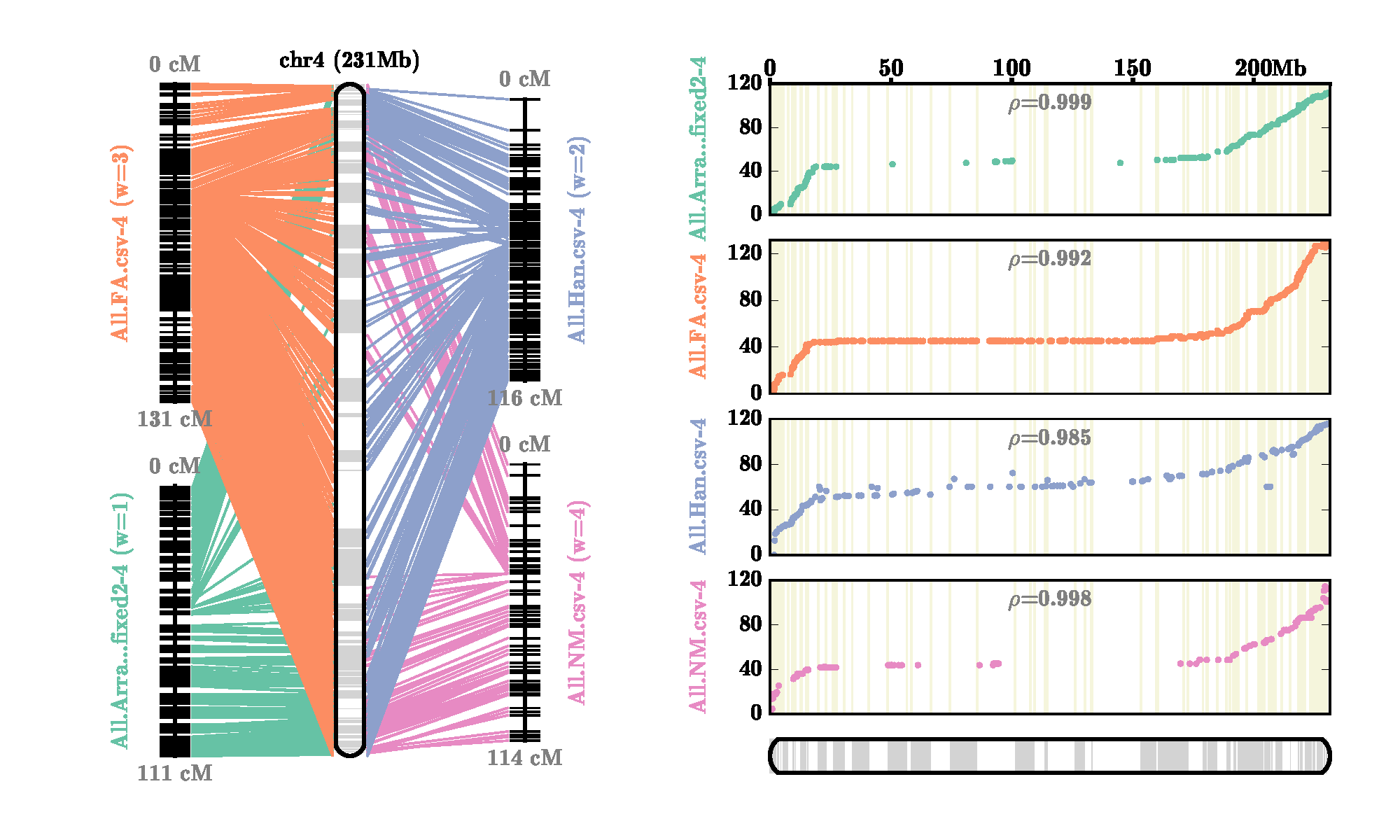


**Supplemental Figure 13 - AllMaps Chromosome 4 Consensus Maps for Pseudomolecule Generation.** Filtered alignment positions from Hill et al. (2015), Hulse-Kemp et al. (2016) and Han et al. (2017) were utilized to generate pseudomolecules using the AllMaps software.


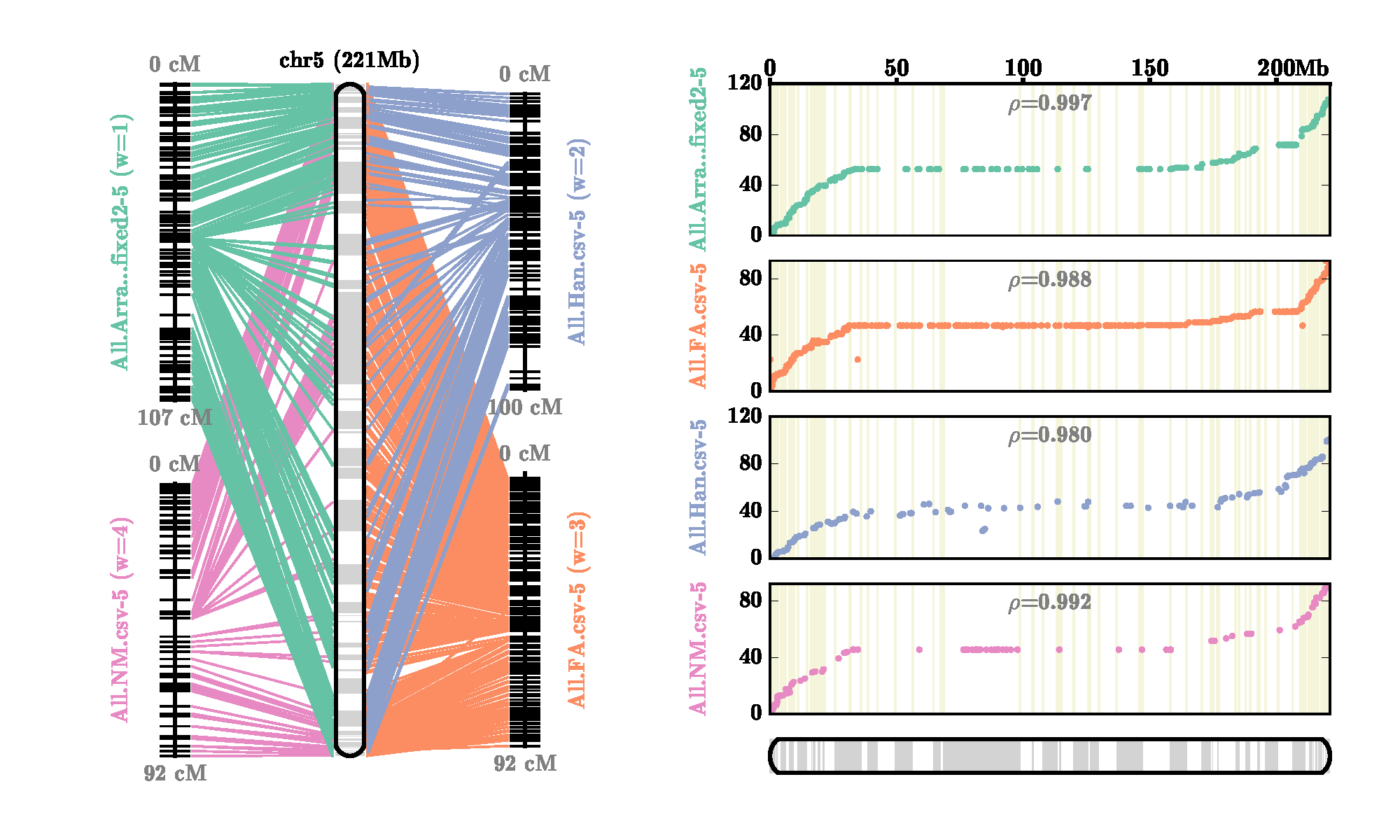


**Supplemental Figure 14 - AllMaps Chromosome 5 Consensus Maps for Pseudomolecule Generation.** Filtered alignment positions from Hill et al. (2015), Hulse-Kemp et al. (2016) and Han et al. (2017) were utilized to generate pseudomolecules using the AllMaps software.


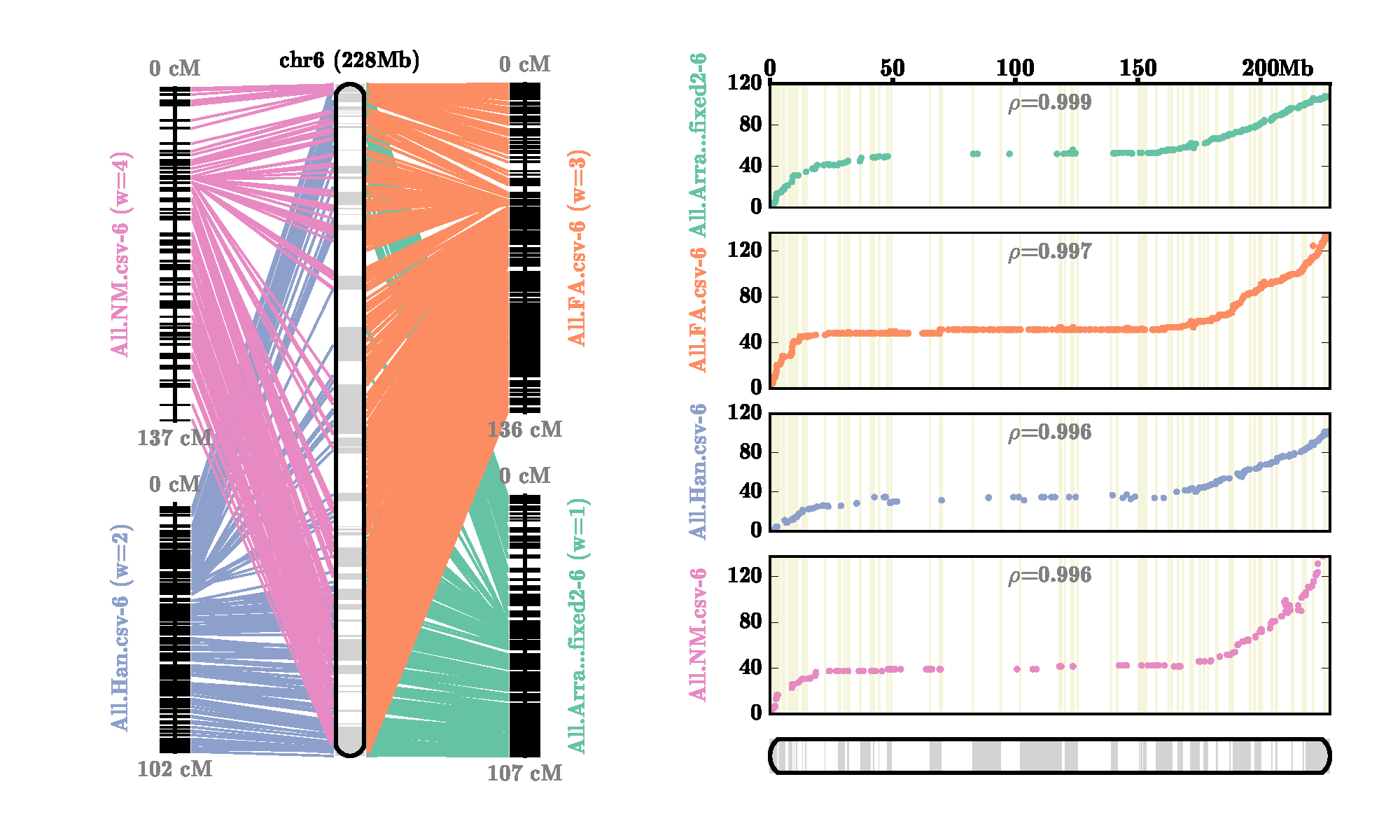


**Supplemental Figure 15 - AllMaps Chromosome 6 Consensus Maps for Pseudomolecule Generation.** Filtered alignment positions from Hill et al. (2015), Hulse-Kemp et al. (2016) and Han et al. (2017) were utilized to generate pseudomolecules using the AllMaps software.


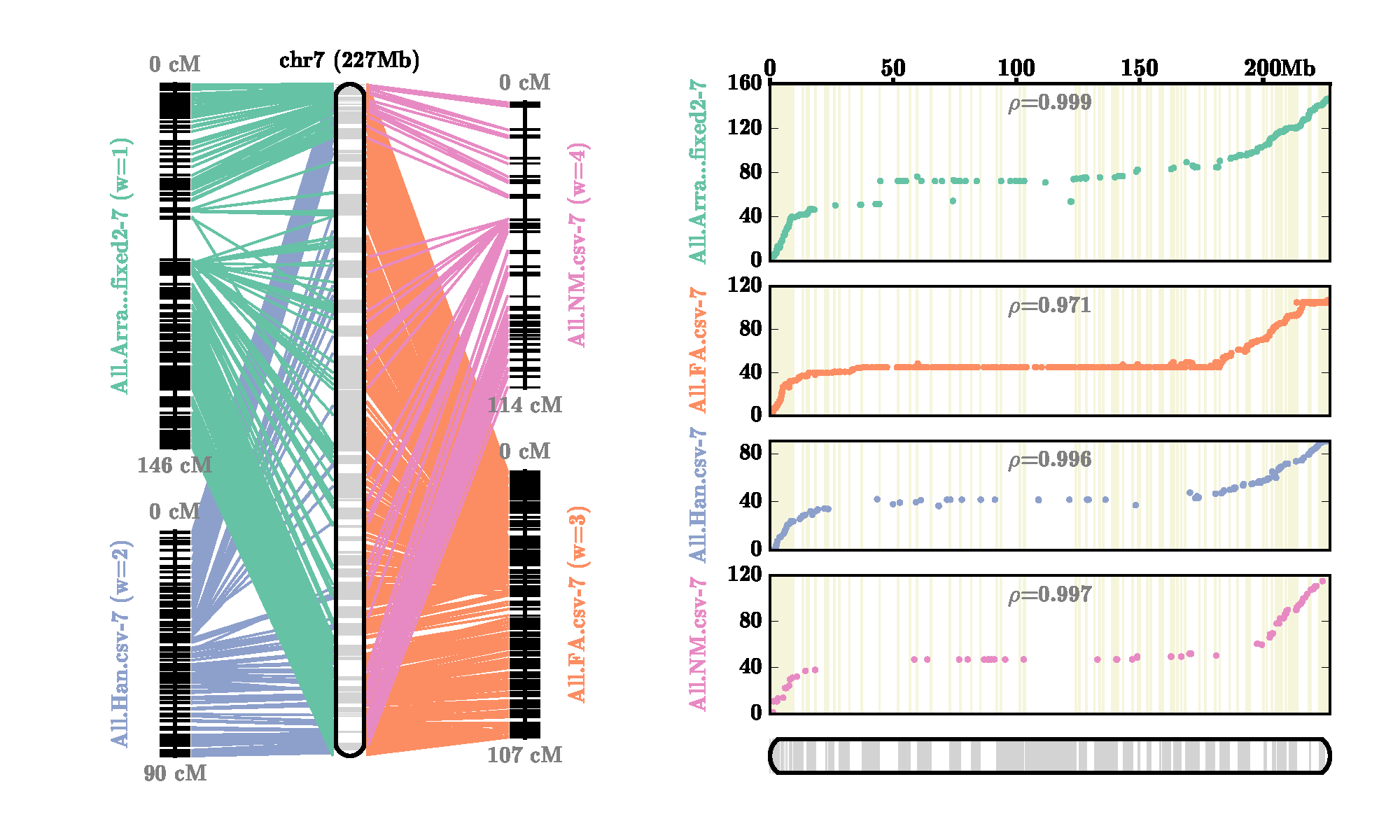


**Supplemental Figure 16 - AllMaps Chromosome 7 Consensus Maps for Pseudomolecule Generation.** Filtered alignment positions from Hill et al. (2015), Hulse-Kemp et al. (2016) and Han et al. (2017) were utilized to generate pseudomolecules using the AllMaps software.


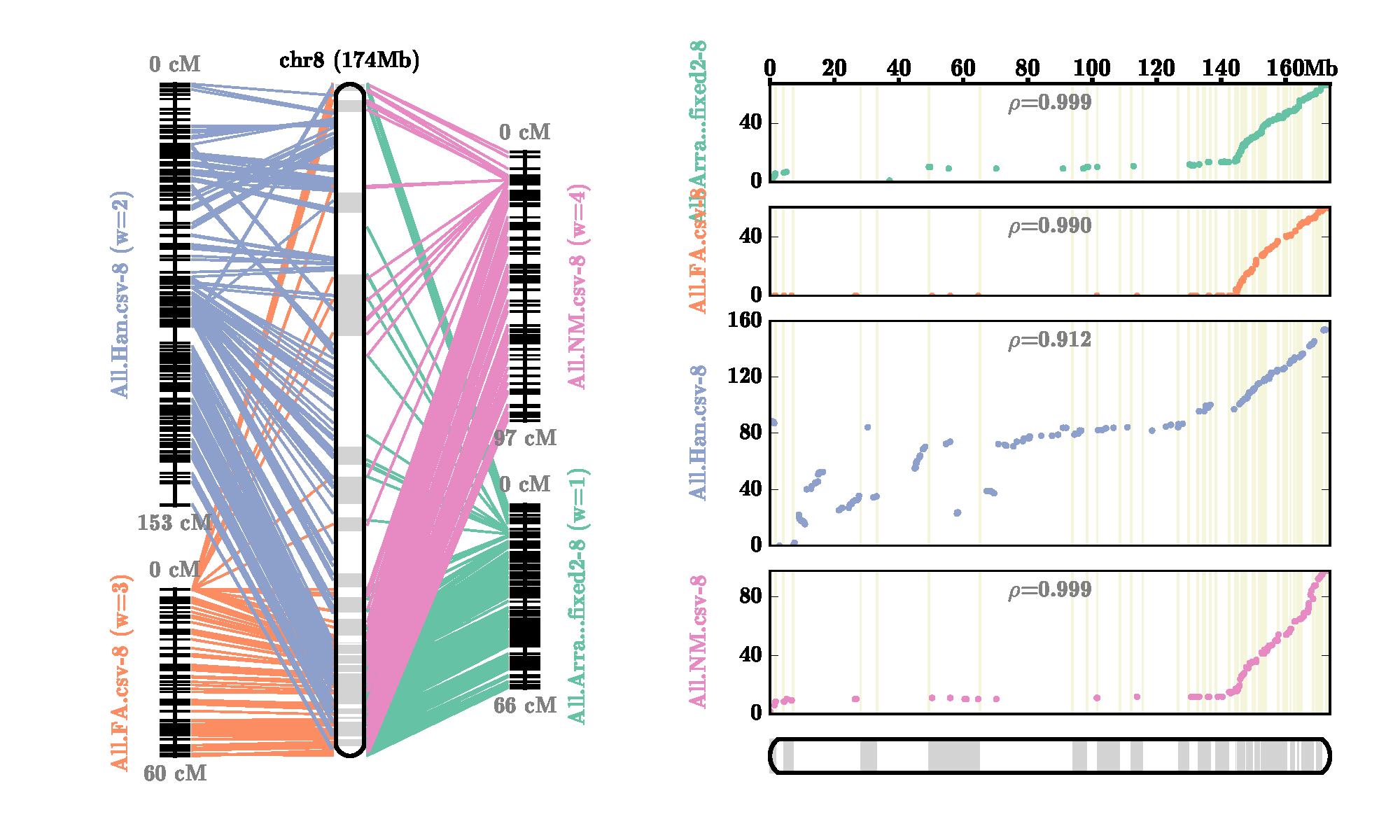


**Supplemental Figure 17 - AllMaps Chromosome 8 Consensus Maps for Pseudomolecule Generation.** Filtered alignment positions from Hill et al. (2015), Hulse-Kemp et al. (2016) and Han et al. (2017) were utilized to generate pseudomolecules using the AllMaps software.


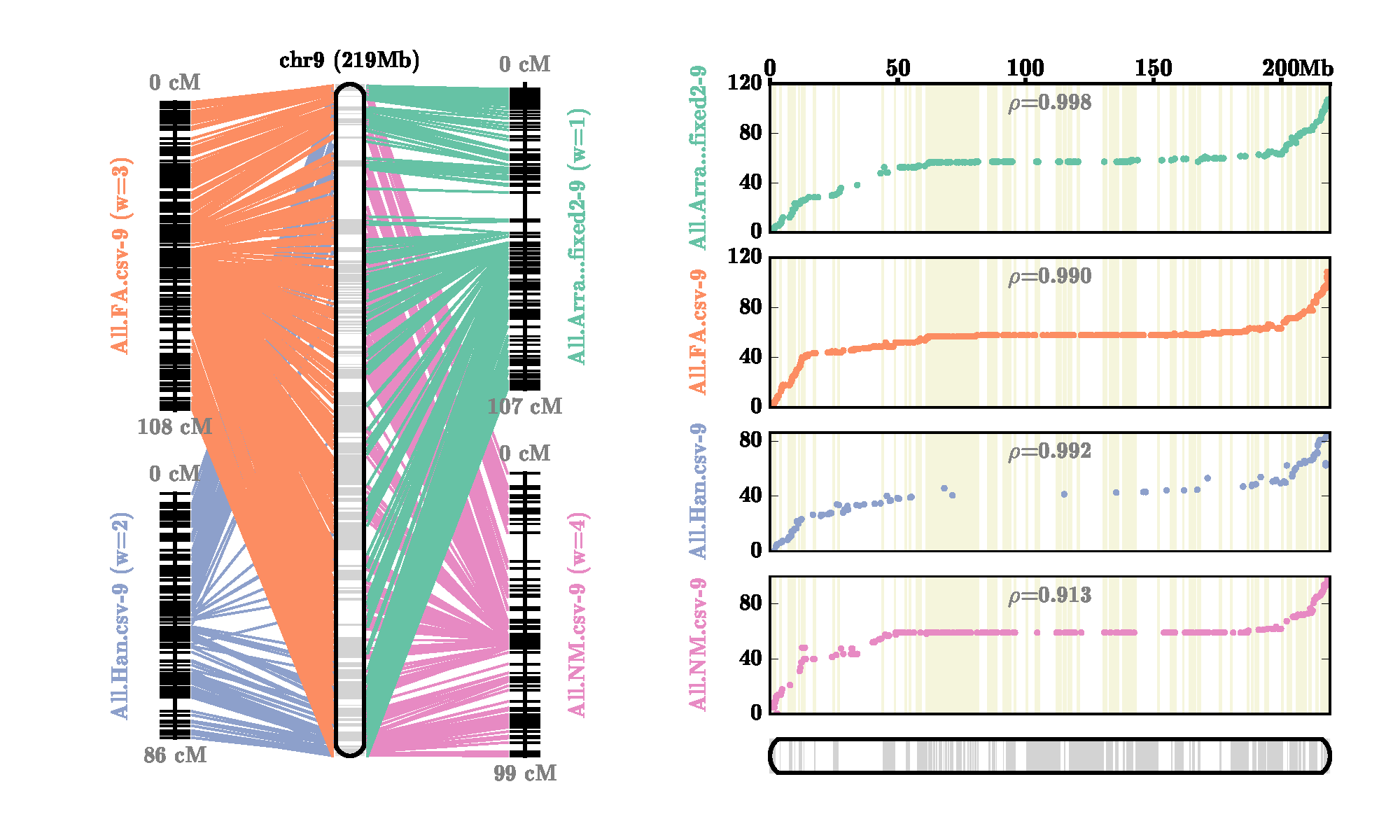


**Supplemental Figure 18 - AllMaps Chromosome 9 Consensus Maps for Pseudomolecule Generation.** Filtered alignment positions from Hill et al. (2015), Hulse-Kemp et al. (2016) and Han et al. (2017) were utilized to generate pseudomolecules using the AllMaps software.


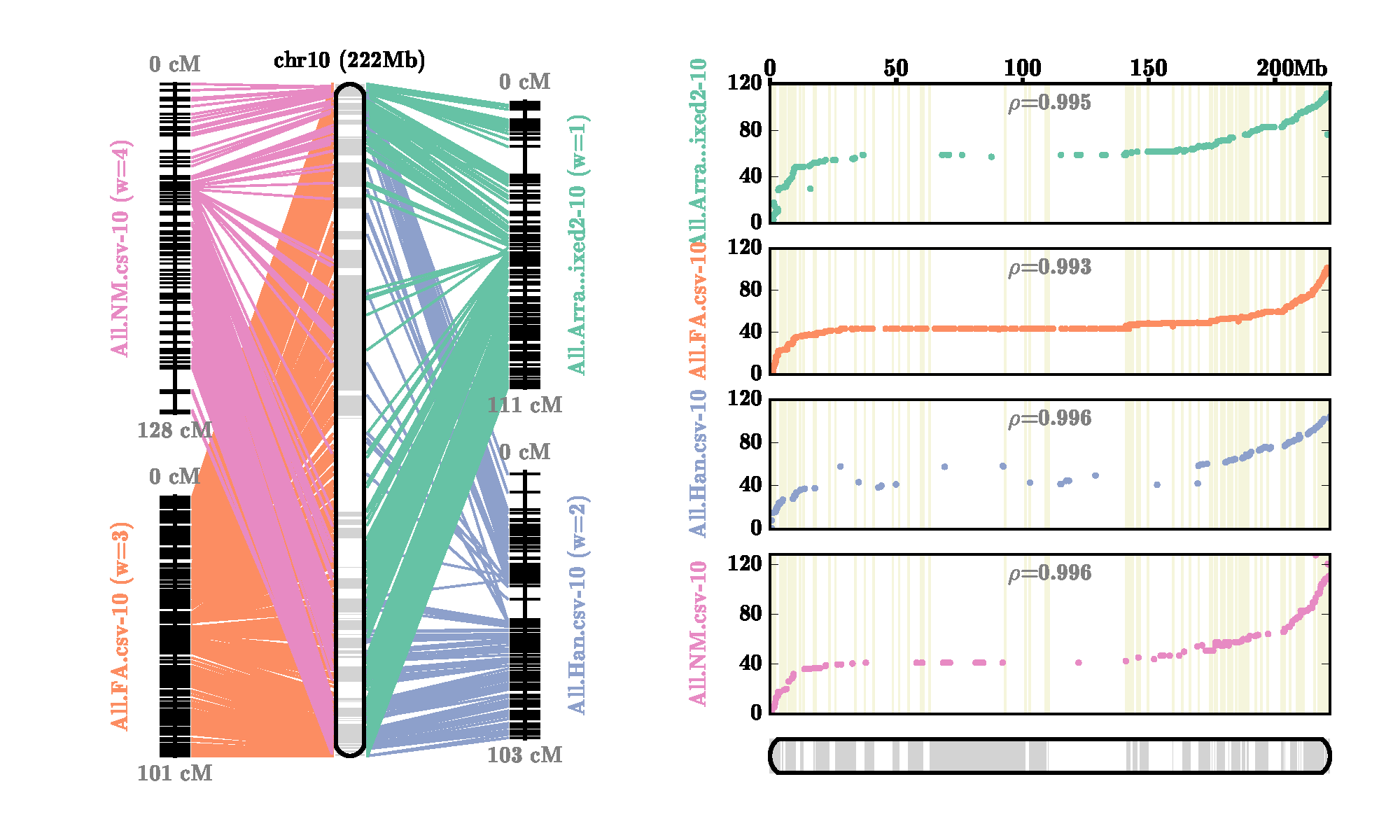


**Supplemental Figure 19 - AllMaps Chromosome 10 Consensus Maps for Pseudomolecule Generation.** Filtered alignment positions from Hill et al. (2015), Hulse-Kemp et al. (2016) and Han et al. (2017) were utilized to generate pseudomolecules using the AllMaps software.


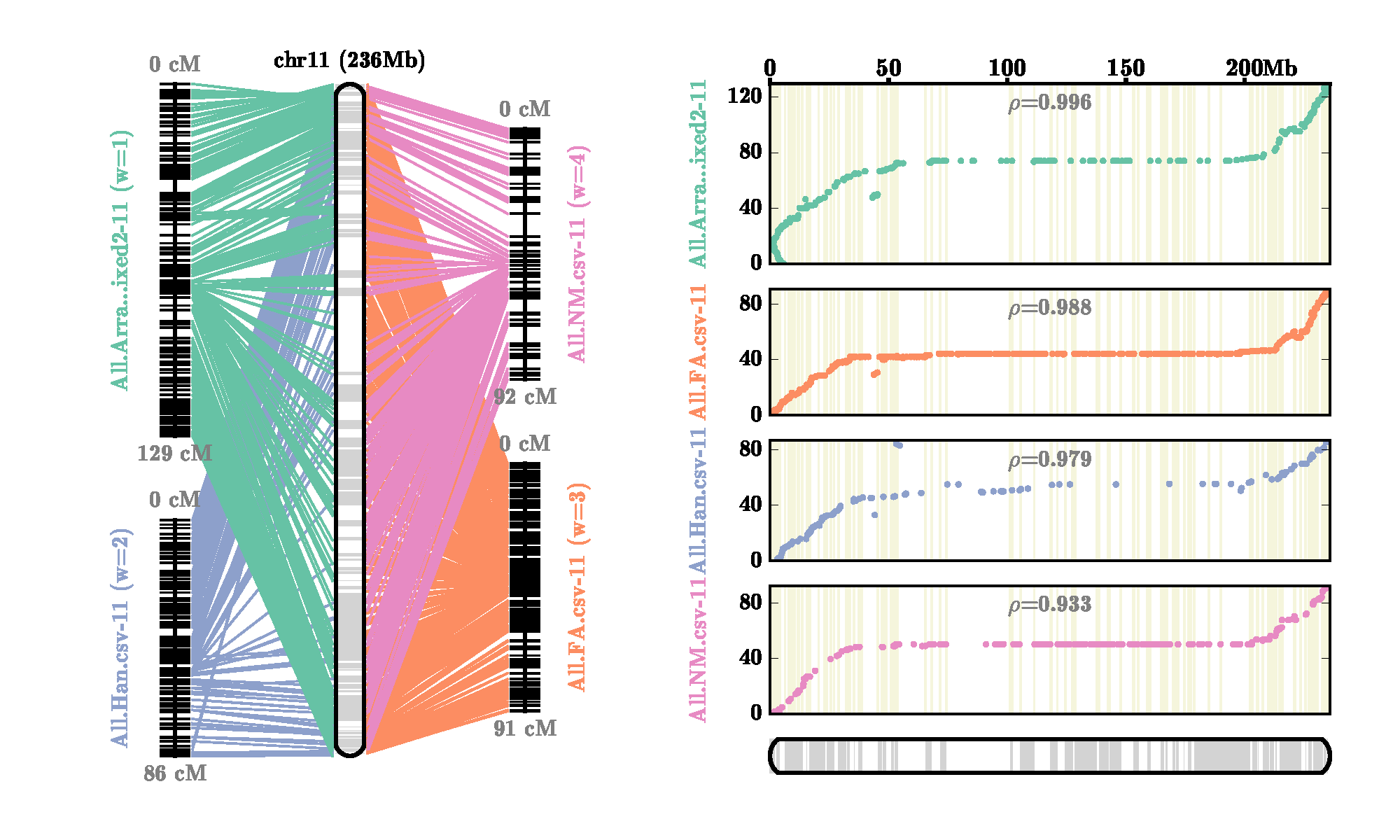


**Supplemental Figure 20 - AllMaps Chromosome 11 Consensus Maps for Pseudomolecule Generation.** Filtered alignment positions from Hill et al. (2015), Hulse-Kemp et al. (2016) and Han et al. (2017) were utilized to generate pseudomolecules using the AllMaps software.


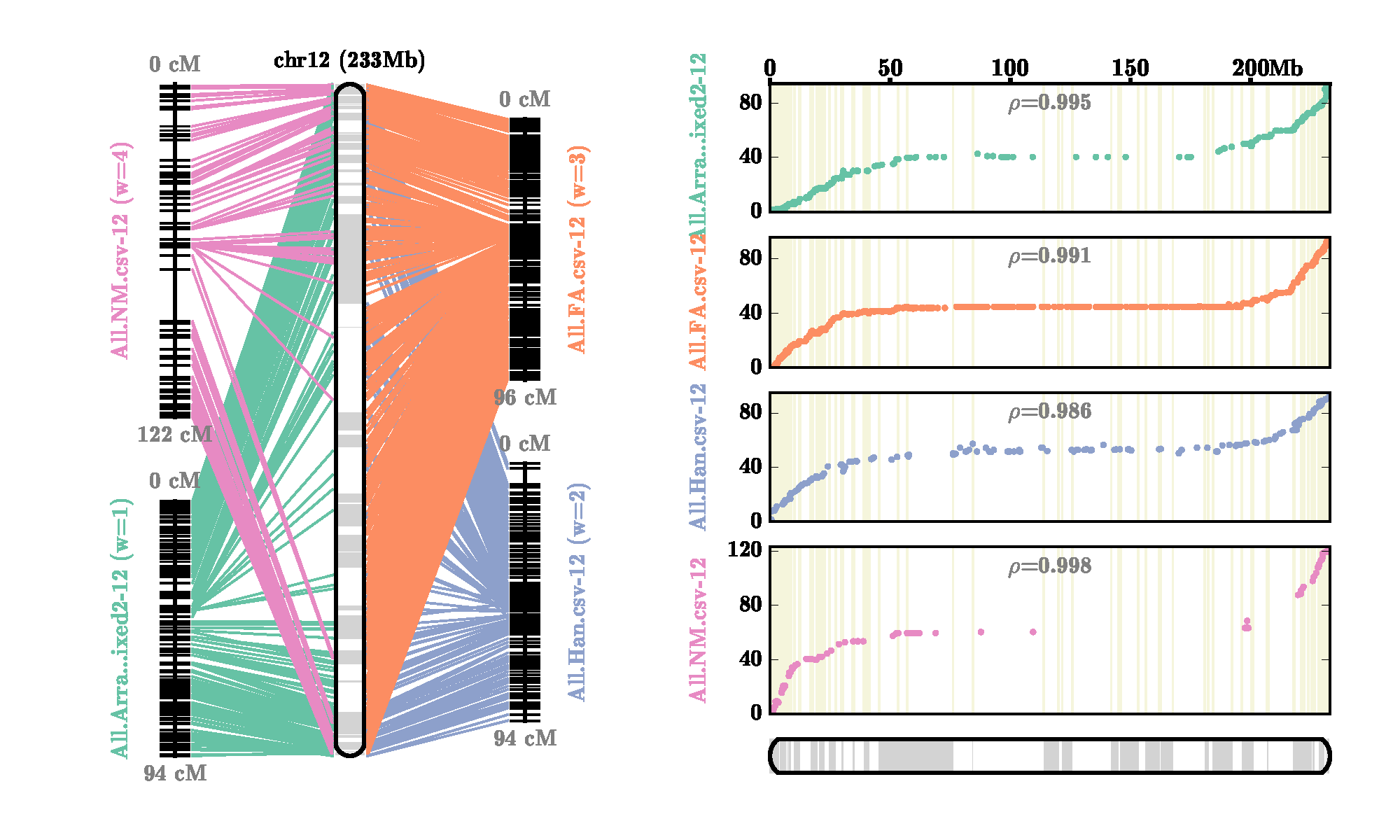


**Supplemental Figure 21 - AllMaps Chromosome 12 Consensus Maps for Pseudomolecule Generation.** Filtered alignment positions from Hill et al. (2015), Hulse-Kemp et al. (2016) and Han et al. (2017) were utilized to generate pseudomolecules using the AllMaps software.


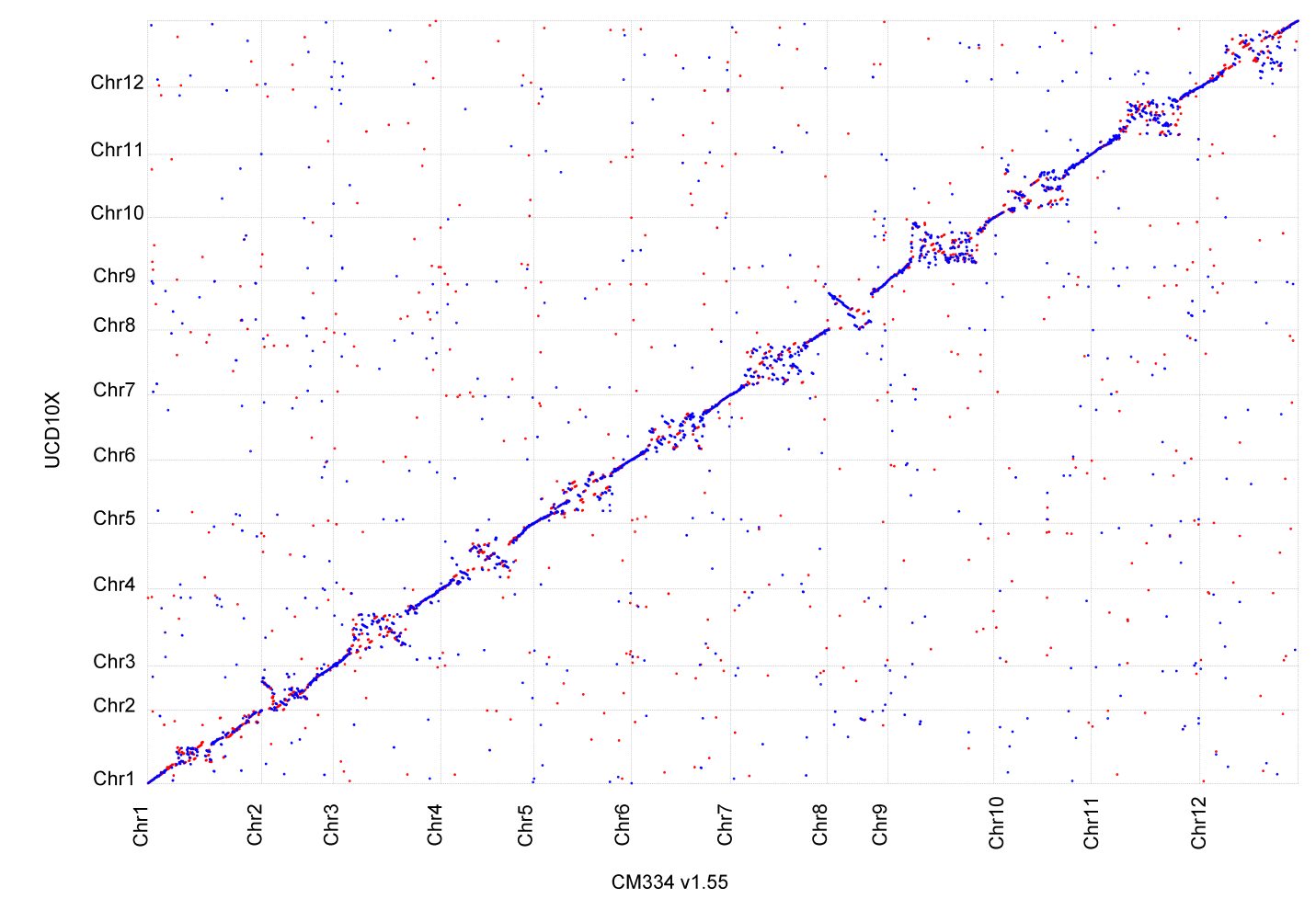


**Supplemental Figure 22** – **Whole Genome Comparison Between UCD10X and CM334 v1.55.** Filtered nucmer alignments for UCD10X (x-axis) and CM334 version 1.55 (y-axis) are shown for alignment lengths of greater than 500 base pairs at greater than 98% sequence identity.


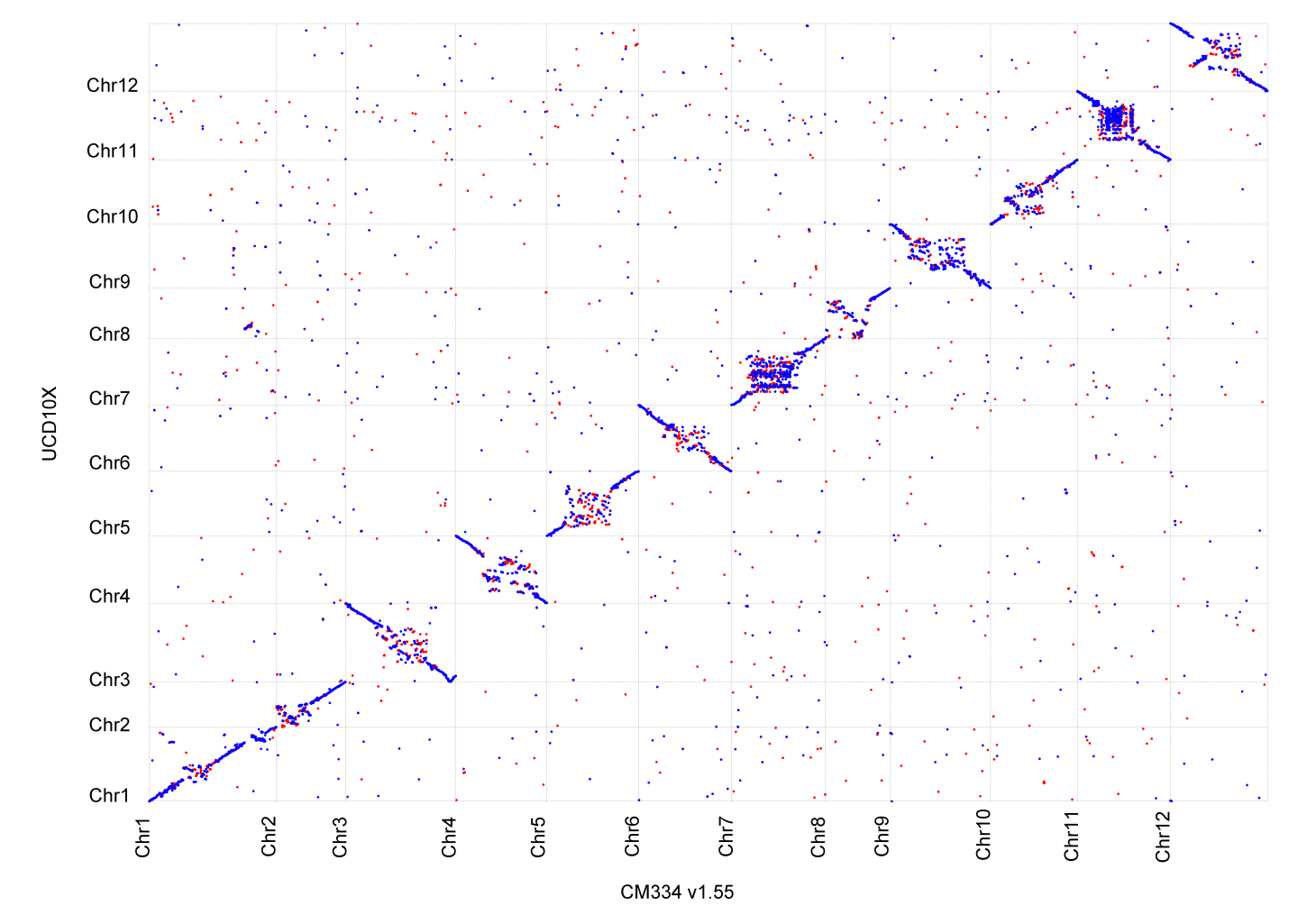


**Supplemental Figure 23** – **Whole Genome Comparison Between UCD10X and Zunla v2.0.** Filtered nucmer alignments for UCD10X (x-axis) and Zunla version 2.0 (y-axis) are shown for alignment lengths of greater than 500 base pairs at greater than 98% sequence identity.

**
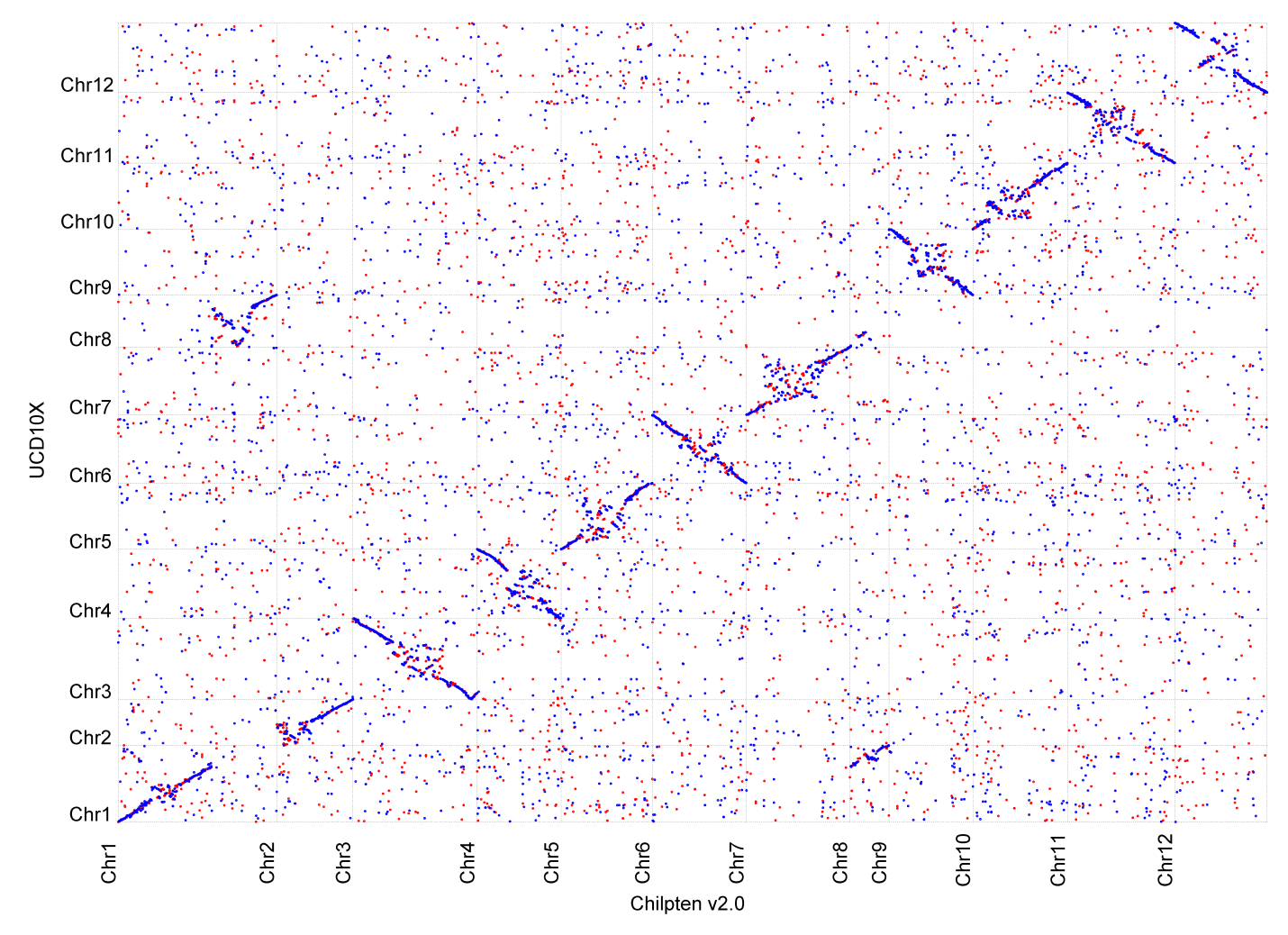
**

**Supplemental Figure 24** – **Whole Genome Comparison Between UCD10X and Chiltepin v2.0.** Filtered nucmer alignments for UCD10X (x-axis) and Chiltepin version 2.0 (y-axis) are shown for alignment lengths of greater than 500 base pairs at greater than 98% sequence identity.


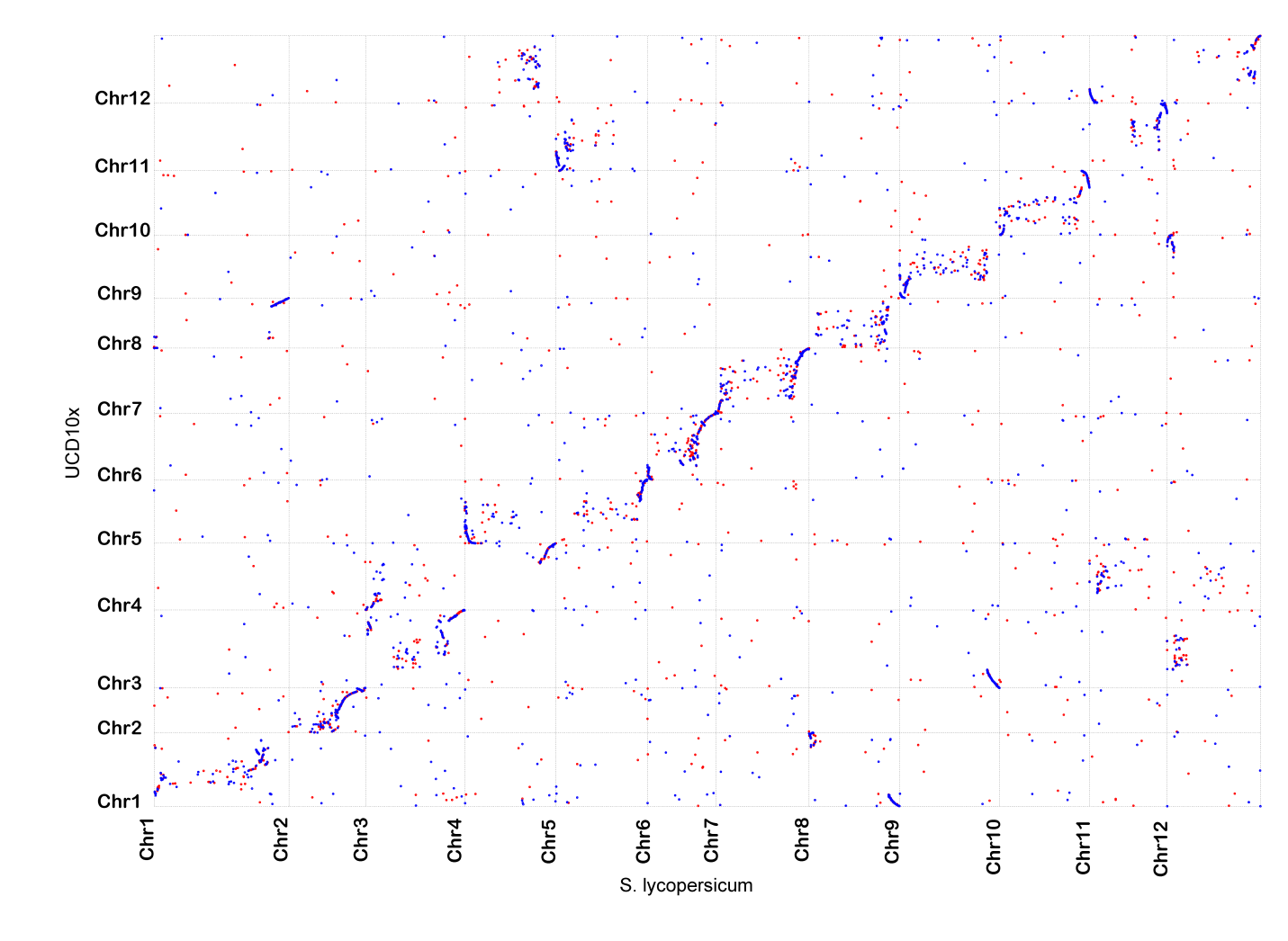


**Supplemental Figure 25** – **Whole Genome Comparison Between UCD10X and Tomato v3.0.** Filtered nucmer alignments for UCD10X (x-axis) and *S. lycopersicum* version 3.0 (y-axis) are shown for alignment lengths of greater than 150 base pairs at greater than 85% sequence identity.
